# Identification of umami peptides and mechanism of the interaction with umami receptors T1R1/T1R3 in pigeon meat

**DOI:** 10.5713/ab.24.0425

**Published:** 2024-10-25

**Authors:** Yue Zheng, Mengnan Cao, Dengyong Liu

**Affiliations:** 1 College of Food Science and Technology, Bohai University, Jinzhou 121013, China; 2 Meat Innovation Center of Liaoning Province, Jinzhou 121013, China

**Keywords:** Molecular Docking, Pigeon Meat, Separation and Purification, Structural Identification, Umami Peptide

## Abstract

**Objective:**

Pigeon meat offer an ideal source for extracting fresh flavor peptides. These peptides not only enhance the taste of food but also have potential health benefits, including providing low-sugar, low-sodium, and low-calorie options for individuals with conditions like diabetes, hypertension, and obesity. Therefore, further research into the pigeon industry holds promise for addressing both economic and nutritional needs.

**Methods:**

To explore umami peptides and their molecular binding mechanisms with umami receptor type 1 member 1 in pigeon meat, an enzymatic hydrolysate product is isolated, analyzed, and subjected to sensory evaluation. Fifteen peptides with high freshness characteristics are separated and identified by ultrafiltration, gel separation, reverse performance liquid chromatography, and nano-liquid chromatography-tandem mass spectrometry (nano-LC-MS/MS).

**Results:**

The molecular docking results show that the amino acid residue Glu128 is a common ligand binding site for all of the fresh-flavored peptides to taste T1R1/T1R3 receptors and it exerts freshness-presenting effects with 15 fresh-flavored peptides through hydrogen bonding, electrostatic interactions, salt bridges, and hydrophobic interactions.

**Conclusion:**

This study provides a theoretical basis and technical support for the subsequent development of flavor peptide products in pigeon meat.

## INTRODUCTION

The industry of pigeon meat in China has developed rapidly in recent years owing to economic growth. Many pigeon farms are being built or expanded, and the stock of breeding pigeons continues to increase [1]. The pigeon industry is developing to scale, industrialize, and intensify its capacity and technology. Meanwhile, consumer requirements for animal protein consumption are constantly increasing, especially for high-quality proteins. Pigeon meat, particularly breast meat, is nutritious because it is rich in high-value proteins and low in cholesterol [2]. However, pigeon meat, in particular leg meat, is characterized by a relatively high fat content and energy value [3]. Pigeon meat protein contains many essential amino acids of the human, and the digestion and absorption rate is 5%, the fat content of pigeon meat is only 3%, lower than other meat. Pigeons are called "animal ginseng" in China. Therefore, pigeon meat is not only nutritious, but also has certain health effects. However, the substance (protein or amino acid) that plays a specific function and the mechanism of action have been lacking more in-depth research.

The rapid spread of the COVID-19 pandemic at the beginning of 2020 and factors such as the backward production modes, breeding level degradation, imperfect industrial chains, and high disease risk restrict the development of the the industry of pigeon meat in China [4,5]. To promote the sustainable development of the industry of pigeon meat, significant steps must be taken, including adjusting the production structure of pigeons meat and developing diversified, unique, and high value-added products.

Fresh flavoring substances are present in a variety of foods, including seafood, meat, and vegetables [6]. It is well known that pigeon, as the fourth most popular poultry after chicken, duck, and goose, is an ideal edible meat tonic because of its taste, high protein, and low fat content and is also an ideal raw material for extracting fresh flavor peptides [7]. Fresh flavor peptides are the current research hotspot in the field of food flavor. Compared with other fresh flavor agents, fresh flavor peptides have good physiological and flavor activities and a high nutritional value, which can increase the intensity of the freshness through a synergistic effect with other substances while giving a delicious taste to food, thus reducing the amount of salt and monosodium glutamate added. Fresh peptides can also provide low-sugar, low-sodium, and low-calorie foods for patients with diabetes, hypertension, and obesity. They also have potential development and utilization value. Therefore, the biological functions of these novel peptides have received increasing attention.

Umami is produced by a combination of free amino acids, nucleotides, peptides, organic acids, and their derivatives with umami receptors, which can improve the overall taste of food. Umami hexapeptides were first discovered in papain-treated beef and were then identified in a variety of foods [8], including pork, chicken soup, fish, and peanuts in plant foods. To date, a variety of G protein-coupled receptors responsible for the recognition of umami substances have been reported, including umami receptors T1R1/T1R3 receptors, mGluR, and CaSR [9,10]. Umami taste is caused by umami compounds that bind to the umami taste receptors T1R1/T1R3. The mode of binding to the receptor may differ for different umami peptides, and the mechanism of flavor formation is complex [11]. Currently, research on the quality of squab meat mainly focuses on the nutritional quality and influence of different preservation and processing methods on the quality of pigeon meat products.

In our previous study, we examined the nutritional and volatile flavor substances in pigeon soup and determined that umami and saltiness were the main flavor characteristics of pigeon soup through sensory and electronic tongue evaluations [12]. However, the umami peptides in pigeon meat and their freshening mechanism have not been thoroughly explored. In this study, peptides were isolated and identified using gel filtration chromatography, reversed-phase high-performance liquid chromatography (RP-HPLC), and liquid chromatography-tandem mass spectrometry (LC-MS/MS). The taste characteristics of the selected umami peptides were determined by sensory evaluation and e-tongue technology. Homology modeling was performed to study the interaction between the identified umami peptides and umami taste receptors T1R1/T1R3, and molecular docking was performed to reveal the molecular mechanism of the flavor umami peptides. The main objective of this study was to identify new umami peptides from pigeon meat and reveal their fresh formation mechanism, which is linked to their receptors T1R1/T1R3 [13].

## MATERIALS AND METHODS

### Materials and chemicals

Materials: King pigeons were purchased from the Jinzhou Yixian Meat Pigeon Farm. American King pigeons were used in the experiment. The adult male pigeons weighed 0.60 to 0.65 kg and were 25 to 30 days old.

The internal organs, heads, and claws were removed after slaughter, and pigeons that weighed 350 to 400 g after slaughtering and gutting were selected. The breast meat of pigeon were selected for the experiment, with 6 pigeons in each group and 3 repetitions in each experiment. Ethical approval was given by the ethics committee of Bohai university with the following number:CFST2023-01-0316.

The enzymolysis protein is made with flavourzyme (SLD-1392; Cangzhou Xiasheng Enzyme Biotechnology Co., Cangzhou, China) and neutral proteases (DISPASE, CAS:9068-59-1; Cangzhou Xiasheng Enzyme Biotechnology Co.). The dextran gel (Sephadex G-15) (Beijing Solarbio Technology Co., Beijing, China). Sugar, salt, and MSGs (Jinzhou Wanwei supermarket, Jinzhou, China). Citric acid (Sigma Aldrich Co., St. Louis, MO, USA), quinine hydrochloride (Shanghai Yuanye Biotechnology Co., Shanghai, China), acetonitrile (chromatographically pure, Merck, Darmstadt, Germany), trifluoroacetic acid (chromatographic pure; Qingdao Jacob Chemical Reagent Sales Co., Qingdao, China), and ultrapure water (Milli-Q preparation) were obtained for use in the following experiments and analyses.

### Preparation of the pigeon meat flavor peptide base

After optimizing the pre-test conditions in the laboratory, 200 g of the pre-treated sample (breast meat), which consisted of a material–liquid ratio of 2:1 (m/v, pigeon meat:deionized water), was homogenized twice for 10 s at 6,000 r/min.

Then, enzyme digestion was conducted. The enzymolysis protein is made with flavourzyme and neutral proteases. A flavor protease (0.1%) was added to the mass of pigeon meat. The enzyme digestion was conducted for 1.5 h at a temperature of 53°C without enzyme inactivation. Then, a neutral protease was added (0.25%) was added to the mass of pigeon meat.

The enzyme digestion was conducted at temperatures of 50°C and 90°C, after which the enzyme was inactivated for 10 min after 2.5 h. The supernatant was used as the pigeon meat flavor peptide base (GR) after the enzyme digestion was removed and centrifuged at 4°C and 10,000 r/min for 15 min. The GR was freeze-dried and stored in the refrigerator at −80°C for subsequent determination of the indicators.

### Determination of the molecular weight distribution of the fresh flavored peptide base

According to Ogsawara’s method for the determination of hydrolyzed soy protein, the molecular weight of the oligopeptides was determined by dissolving 1 mg of a lyophilized sample of a fresh peptide crude extract in 1 mL of the mobile phase, centrifuging it (10,000 r/min for 15 min), and extracting the supernatant for determination [14].

### Isolation and purification of fresh flavor peptides

#### Ultrafiltration separation

The supernatant was collected and graded by ultrafiltration. The samples were separated by ultrafiltration using an ultrafiltration membrane with a molecular weight cut-off of 3 kU at 4°C. A fraction with a molecular weight of less than 3 kU was collected and named UF-GR, which was concentrated by rotary evaporation at 80°C before being freeze-dried and stored in a refrigerator at −80°C for spare use.

#### Gel chromatographic separation and purification

UF-GR was dissolved in ultrapure water (20 mg/mL), filtered through a 0.45-μm membrane, and eluted on a packed gel filtration chromatography column (packed with Sephadex G-15) using ultrapure water as the mobile phase with a flow rate of 1 mL/min, sample volume of 3 mL, and detection wavelength of 220 nm. The UV detection wavelength was 220 nm and the component collection time was set to 3 min for each tube. Each separated peak component was collected in groups, concentrated, and freeze-dried under vacuum before being placed in the refrigerator at −80°C for backup, and the samples collected from different peaks were named G1, G2, etc. accordingly. Then, the collected components were evaluated for their sensory qualities.

#### LC-MS/MS identification of fresh flavor peptides

After gel chromatography, purified pigeon oligopeptide samples were desalted using a C18 desalting column.

Oligopeptide sequence identification was performed for the desalted samples, nano-high-performance liquid chromatography-tandem mass spectrometry (nano-HPLC-MS/MS) analysis was performed, and the samples were analyzed using an LC-MS/MS instrument equipped with an online nanospray ion source. The complete system used was the OExactiveTM MS (Thermo Fisher Scientific, Waltham, MA, USA) coupled with an EASY-nanoLC1200. The volume of the test sample was 1 μL (model and specifications of the analytical column: Acclaim PepMap C18, 75 μm, 25 cm). The gradient separation of the sample was conducted within 60 min, and the column flow rate, column temperature, and electrospray voltage were maintained at 400 nL/min, 40°C, and 2 kV, respectively. The gradient started in phase B (80% ACN with 0.1% FA), increased nonlinearly to 60% at 46 min, increased to 100% at 4 min, and was maintained for 10 min.

The MS was operated in the data-dependent acquisition mode to realize automatic conversion between MS and MS/MS acquisition. The MS parameters were set as follows: scanning range (m/z) of 200 to 1,800, resolution of 70,000, AGC target of 3e6, and upper limit of injection time of 60 ms. The HCD-MS/MS parameters were as follows: resolution of 17,500, AGC target of 5e4, maximum injection time of 80 ms, collision energy of 27, and dynamic rejection time of 20 s.

Tandem mass spectra were analyzed using the PEAKS Studio version 10.6 (Bioinformatics Solutions, Inc., Waterloo, ON, Canada). PEAKS DB for uniprot-Trichiurus lepturus, uniprot-Trichiuruslepturus+Helianthusannuus, and uniprot-Trichiurus lepturus+Helianthus annuus+Cicer arietinum (version202201,76207 enteries) were subjected to a database search with the enzyme digestion set. The search library parameters were set to an allowable mass error of 0.02 Da for fragment ions, mass error of 7 ppm for parent ions, and variable modifications of 15.99 for oxidation (M), 0.98 for deamidation (NQ), and 42.01 for acetylation (Protein N-term), with protein and peptide cardinalities of log logarithm (to the base 10) p≥0 and p≥20, respectively, as well as at least one peptide with specificity.

#### Fresh peptide activity prediction

The potential bioactivities of the identified peptides were predicted using the flavor-presenting amino acid database, BIOPEP (http://www.uwm.edu.pl/biochemia/index.php/pl/biopep) for the potential bioactivities of the fresh peptides, prediction of the flavor profiles of the identified peptides by LC-MS/MS, and selection of fresh peptides and their fragments with fresh flavor profiles.

#### Screening of fresh flavor peptides by molecular docking technology

The BIOPEP database was used to predict the peptides with higher fresh taste activities, and further molecular docking of fresh taste-active peptides with fresh taste receptor T1R1/T1R3 was conducted using the Discovery Studio 2017 software. The peptides were screened and analyzed based on their docking interaction, total docking, and lower docking energies and ability to bind to the fresh taste receptor T1R1/T1R3.

The 3D structure of the pigeon meat fresh flavor peptide was constructed using the molecular docking software (Discovery Studio 2017), followed by energy minimization using the minimization ligand module. Molecular docking of the T1R1/T1R3 receptor model constructed with the peptide was performed using the CDOCKER protocol, and the peptide was screened according to the receptor versus ligand docking energy screening peptides.

#### Evaluation of sensory qualities

A total of ten healthy, non-smoking, non-taste/odor-impaired panelists, specifically five males and females that were 20 to 30 years old, were recruited for the sensory experiments. All panelists had at least 1 year of experience in sensory evaluation and agreed to participate in the sensory tests of the current study. The panel was trained to perform sensory evaluations of the test and standardized samples. The samples used in the evaluations were filtered with a 0.22-μm filter membrane presenting them to the panelists.

Scale method (such as 5-point scale method) is used to analyze the flavor profile of food, and then the key flavor components are explored by means of subtracting and adding experiments [15]. The premise of the elimination experiment is to quantify the potential flavor components and prepare the artificial recombination solution according to the concentration of each component, and then remove one or a group of flavor components successively and present it to the sensitivity officer, who is asked to judge the change of the flavor profile of the recombination solution after the removal of components by three-point test method.

First provide 4 to 6 samples - familiarize the sensory officer with these samples; second, give the same as the first group of multiple samples (8 to 10), select the same as the first group of samples for training. Training steps: Obtain a general vocabulary or score sheet based on the description of the participants; Rearrange, organize, and form a comprehensive and clear glossary or scoring sheet; Choose appropriate references for these words or ratings. During the training, each sample was tasted 2 to 3 times, described or scored (each sample was repeated 6 times, with a 10-minute rest in between).

The enzymatic stock solution, ultrafiltration, and gel chromatography lyophilized fractions (3 groups of samples, 6 replicates per sample) were prepared as 100 mL each of a 1 mg/mL solution and scored from 0 to 7, with 0 representing no taste and 7 indicating significant taste. Citric acid (0.430 mg/mL), sucrose (5.76 mg/mL), quinine sulfate (0.0325 mg/mL), table salt (1.19 mg/mL), and monosodium glutamate (0.595 mg/mL), which represented sour, sweet, bitter, salty, and fresh flavors, respectively, were used as the evaluated standards. The standards at these concentrations were identified as a score of 4. Each component was scored in comparison with the standard.

### Data analysis

The peptides were predicted using the "Sensory Peptides and Amino Acids" module of the bioactive peptide database, BIOPEP, and the molecular docking of the flavored peptides to the receptor T1R1/T1R3 was performed using the Discovery Studio 2017 software. The docking binding sites, docking interaction force, total docking energy, and docking interaction energy were analyzed [16]. Plots were constructed using the Origin 2018 software.

## RESULTS and DISCUSSION

### Molecular weight distribution of the polypeptide crude extract

Studies have shown that the length of the peptide chain and molecular weight play different roles. Functional peptides in the cosmetics industry are mainly small molecular weight peptides, and peptides with low molecular weights in food have an important impact on the flavor [14]. The length of the peptide chain, that is, the molecular weight of the peptide, affects the flavor characteristics of the peptide, and the molecular weight of the peptide with umami is generally below 1 kU [17]. In this study, we determined the molecular weight of the crude polypeptide extract. The molecular weight of the enzymatic polypeptide crude extract was divided into five categories: above 3,000, 10,00 to 3,000, 500 to 1000, 189 to 500, and below 189 Da (Table 1).

The molecular weights of the majority of the peptides in the crude extract were below 3,000 Da, accounting for 88.84%, whereas the peptide content with a molecular weight above 3,000 Da was the lowest at approximately 11.16%. The molecular weight of the enzymatic hydrolysis polypeptide crude extract was mainly distributed in the range of less than 3,000 Da. Studies have also shown that umami peptides are a class of oligopeptides with molecular weights less than 3,000 Da [18,19]. Therefore, in our study, peptides with molecular weights below 3,000 Da were used as the basis for screening pigeon meat umami peptides, and the enzymolysis solution was ultrafiltered, separated, and purified.

### Ultrafiltration and gel chromatography separation

Ultrafiltration is based on the pressure difference between the two sides of the ultrafiltration membrane and selection of substances with different molecular weights. Through membrane screening, the solute, which is larger than the membrane aperture in the sample, is intercepted, and the solvent, which is smaller than the membrane diameter, passes through the membrane to achieve the purpose of fractionation. Owing to the advantages of simple equipment, controllable conditions, a non-damaging molecular activity, a high selectivity, and a low energy consumption, membrane separation technology is widely used for the preliminary separation of polypeptides. The authors studied umami peptides and determined that they are a class of oligopeptides with molecular weights less than 3,000 Da, so we chose components with molecular weights less than 3,000 Da for separation [20].

**Figure 1:**
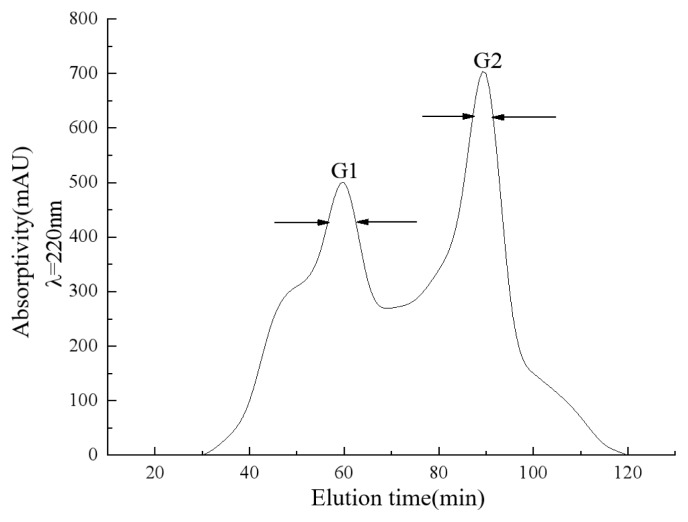
Results of umami peptide gel chromatography separation. The peptide components were separated by gel filtration chromatography. Two peaks were identified as G1 and G2. The peak area of G2 was larger than that of G1, indicating that the relative content of G2 was higher.

The peptide component of the UF-GR lyophilized powder after ultrafiltration was prepared in a solution with a concentration of 20 mg/mL. An AKTA Purifier100 protein purifier was used for purification. As shown in Figure 1, the peptide component UF-GR was separated using a SephadexG-15 packing column. After elution for 120 min, two peaks were detected at 220 nm, named G1 and G2. The peak area of G2 was larger than that of G1, indicating a higher relative content of G2 [21].

**Figure 2:**
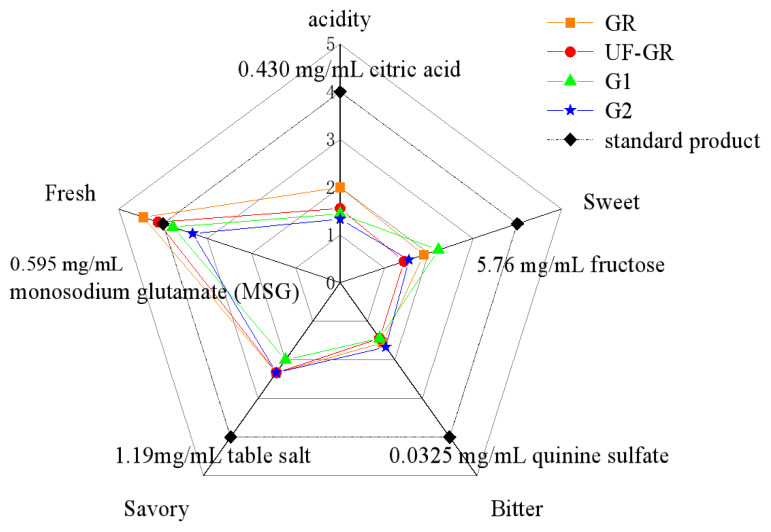
Sensory evaluation radar charts for different components. GR, crude extract of pigeon meat umami peptide without enzymatic hydrolysis; UF-GR, umami peptide components with molecular weight less than 3 kU; G1 and G2, two components of UF-GR were obtained by gel filtration chromatography.

As shown in Figure 2, the taste profiles of the GR, UF-GR, G1, and G2 components are approximately the same. Umami features were the most prominent, followed by the sour, salty, sweet, and bitter features. The results showed that the purified components with higher umami values also had obvious characteristics of sweetness and umami, which may be caused by the mutual promotion and synergistic interactions between umami, sweet, and umami substances [22]. After the separation and purification of the GR, the umami intensity decreased, and the umami intensity from large to small was GR>UF-GR>standard substance>G1>G2. The umami values of the GR and UF-GR components were higher than those of the standard substances, while G1 and G2 of the GC-lyophilized components were lower than those of the standard substances. After purification, the umami intensity of the G1 was higher than that of G2, and the bitterness value of G1 was lower than that of G2. The G1 component was selected for further separation and identification to obtain the key umami peptide of a higher purity.

### Identification of umami peptides by nano-HPLC-MS/MS

MS is the most commonly used method for identifying umami peptides. The principle is to use electric and magnetic fields to separate ions according to the mass-charge ratio for detection and analysis. The method can identify the amino acid sequence and molecular weight of umami peptides efficiently and accurately. Nano-HPLC-MS/MS is an important tool in the field of proteomics. Compared with traditional LC-MS/MS, nano-HPLC-MS/MS can analyze peptide mixtures with a limited number of samples [23].

**Figure 3:**
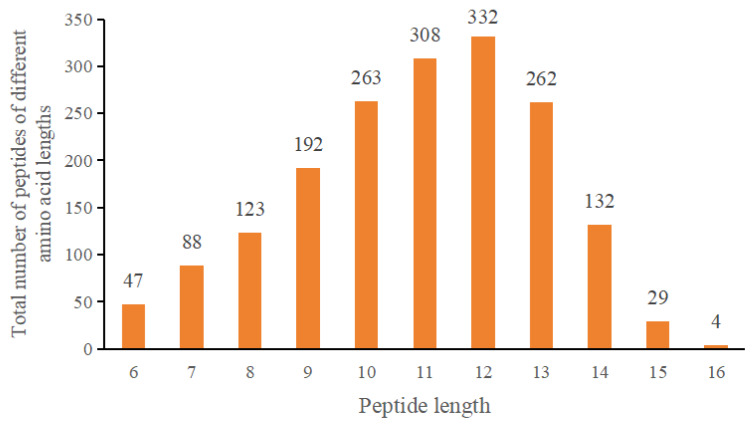
G1 component LC-MS/MS identified peptide distribution. The longer the amino acid sequence of the peptide segment, the greater the influence on the taste of the peptide. From the perspective of peptide length, the proportion of peptides with 9 to13 amino acids was 76.24%, the proportion of peptides with 6 to 8 amino acids was 14.49%, and the proportion of peptides with 14 to 16 amino acids was 9.27%. LC-MS/MS, liquid chromatography-tandem mass spectrometry.

To further explore the composition of umami substances in pigeon meat, nano-HPLC-MS/MS was used to isolate and identify peptides with umami activity in G1. The amino acid sequence and molecular weight of the G1 peptide were identified and analyzed using nano-HPLC-MS/MS. As shown in Figure 3, 1780 peptide segments were identified by MS and were divided into 11 types according to the lengths of the peptide segments. The results were as follows: 47 hexapeptides, 88 heptapeptides, 123 octapeptides, 192 peptides, 263 decapeptides, 308 undecapeptides, 332 dodecapeptides, 262 trideceptides, 132 tetradeceptides, 29 pentapeptides, and four hexadeceptides. The molecular weights of the peptides were in the range of 367.2144 to 1,499.835 Da. The total number of peptide segments and the data of different length of peptide segments can be calculated from Figure 3, the maximum proportion of peptides with 9 to 13 amino acids was 76.24% (192+263+308+332+262/1,780), the proportion of peptides with 6 to 8 amino acids was 14.49% (47+88+123/1,780), and the minimum proportion of peptides with 14 to 16 amino acids was 9.27% (132+29+4/1,780). Studies have shown that the characteristics of umami peptides are affected by many factors, such as the peptide length, structure, and amino acid composition [24], and the length of the peptide sequence is proportional to the taste value. Peptides with umami flavor mainly consist of 2 to 15 amino acids [25]. The G1 component was identified by LC-MS/MS, and its peptide segment was mainly consisted of 9 to 13 amino acids, which is consistent with the results of previous studies. Preliminary results showed that a large number of peptides were identified by MS, and the taste characteristics of 1,780 identified peptides were predicted using the BIOPEP database to further identify peptides that had a strong umami taste.

### Prediction of the umami peptide activity

The BIOPEP database is an online software that can analyze the biological activity of small-molecule peptides. Through the "Sensory Peptides and Amino Acids" module prediction in the database, the potential taste profile of the peptide sequence can be determined, and the relationship between the molecular structure of the peptide and its sensory properties can be analyzed to establish the potential sensory properties of the peptide. To further understand the potential taste properties of peptides, most studies on peptide functions have used the BIOPEP database to predict the taste properties of peptides.

The frequency of the occurrence of bioactive fragments in a peptide is the standard for determining the umami activity of the target peptide. Based on the frequency of the occurrence of umami peptides, the result was calculated using the formula A = a/N, where a represents the number of fragments with specific taste activities in the target peptide sequence and N represents the amino acid residue in the target peptide. A total of 153 peptides with umami activity were screened using a comparative database. The higher the umami activity value, the more likely the target peptide is to have an umami taste. Therefore, a relatively high threshold value of 0.5 was set as the screening condition, and 153 peptides with umami activity were identified. Table 2 shows 15 selected peptides with strong umami characteristics (TKQEYDEAGPS, ITKQEYDEAGPS, IHAEAPEFVEMS, TDADAMKYT, LTDADAMKYT, LNAEGSVEEVFQQ, KAFEEAEK, IDDLEDELYAQK, ISEELDHALNDMT, YQSEEDRKNIL, IYGAEARKESRG, IKWADAGAEYVVE, TDKLKEAETRAE, LTYQSEEDRKNI, LGDEETVRKAMEA).

The peptide with the highest frequency of active umami fragments was IDDLEDELYAQK, followed by KAFEEAEK and IKWADAGAEYVVE, and the peptide with the lowest frequency of active umami fragments was ITKQEYDEAGPS. The most frequent bitters were KAFEEAEK and IDDLEDELYAQK, followed by LNAEGSVEEVFQQ and IKWADAGAEYVVE. The least bitter peptide was LTYQSEEDRKNI. The peptide with the highest frequency was IKWADAGAEYVVE, and that with the lowest activity was ISEELDHALNDM.

Taste characteristics of umami peptides are related to their amino acid composition. The existence of umami-related amino acid peptides contributes to the production of the umami flavor. In particular, aspartic acid (D) and glutamic acid (E) are important amino acids in umami peptides. The peptides identified in Table 3 all contained D or E. However, some also contained histidine (H), proline (P), and arginine with bitterness. For example, two of the seven umami peptides identified by Liu et al [17] did not contain D or E, and the four umami peptides identified from the hydrolysate of silkworm pupae did not contain D or E. Thus, it has been suggested that umami amino acids (D and/or E) may not be indispensable for the composition of umami peptides. In addition, amino acids with bitter taste characteristics, such as valine, arginine, leucine, isoleucine, phenylalanine, and P, also exist in the amino acid peptide segment of umami flavor peptides, and the umami flavor peptides discovered by Dang contained H and V.

### Molecular docking of umami peptides with T1R1/T1R3

Molecular docking is the process of matching two or more molecules using energy to obtain the best binding mode between them [26]. Molecular docking is a computer simulation method used to study the interactions between small-molecule ligands and receptors. The synergistic effect of T1R1/T1R3 with the umami peptides and sodium glutamate has been studied using molecular docking technology, and the mechanism of the synergistic effect between umami peptide and sodium glutamate has been confirmed [27]. To further study the mechanism of the interaction between the peptides and T1R1/T1R3, a good model of T1R1/T1R3 was established in the laboratory. Fifteen peptide sequences with umami activity were simulated using DS software with receptor molecules, the optimal docking parameters and docking energy were selected, and the peptides that could bind to the receptor with low energy were screened.

Molecular docking was used to perform molecular simulation screening of 15 peptides. Table 3 shows that all identified peptides docked with T1R1/T1R3. The total docking energy of R-15 was the lowest at −241.395 kcal/mol. The greater the negative value of the standard free energy charge, the better the binding affinity between the ligand and receptor. The docking energy of R-1 was −92.6761 kcal/mol, the negative value of the standard free energy charge was the smallest, and the binding to the receptor was unstable. As shown in Table 3, the peptide chain length of R-15 and R-12 is the same. The total energy of R-15 was −241.395 kcal/mol and its docking energy was the lowest, while the total energy of R-12 was −180.501 kcal/mol. Therefore, the length of the peptide chain did not correlate with the docking energy between the umami peptide and umami receptor. The binding site and interaction forces between the peptide chain and receptor should also be considered to explore the novel mechanism of umami peptides and receptors.

**Figure 4:**
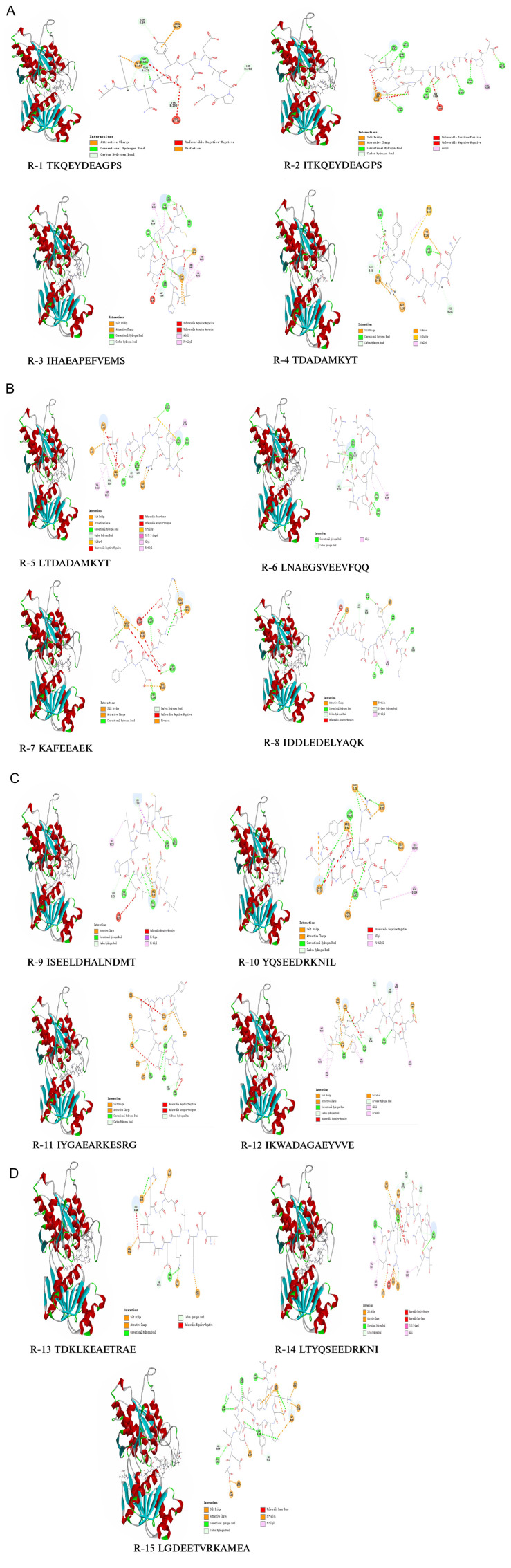
Umami peptide docking results with umami receptor T1R1/T1R3. Green represents hydrogen bonds, yellow represents salt Bridges, orange represents electrostatic interactions, pink-purple repre-sents carbon-hydrogen bonds, pink represents pi-pi T-shapes, light blue represents pi-alkyl, and red represents reaction forces. The amino acid residues of T1R1/T1R3 receptor are bound to each other mainly by electrostatic interaction, hydrogen bonding and salt bridge.

The 2D and 3D drawings of the docking results of the umami peptide and T1R1/T1R3 are shown in Figure 4. Analysis of the binding site of the peptide and T1R1/T1R3 revealed that the binding site mainly involves four forces: salt bridges, hydrogen bonds, hydrophobic interactions, and electrostatic interactions. As shown in Figure 4, Glu128 is the binding site for all peptide chains and receptors. As shown in Figure 4, the binding sites of the amino acid residues and receptors are mainly connected by green hydrogen bonds, yellow salt bridges, and orange electrostatic interactions. Arg34 in umami peptides R-4, R-5, R-6, and R-12 mainly interacted with the receptor through hydrogen bonding, whereas Asp196 in umami peptides R-12, R-14, and R-15 mainly bound to the receptor through electrostatic interactions. The amino acid residues of the T1R1/T1R3 receptors bind to each other mainly through electrostatic interactions, hydrogen bonding, and salt bridges. Dang et al [28] determined that hydrogen bonds and electrostatic interactions are important forces in the interactions between the acceptor and ligand.

To further explore the mechanism of action between umami peptides and their receptors, the binding sites of 15 types of umami peptides and their receptors were analyzed. The results are shown in Table 4, during the interaction between the umami peptides and receptors, there were 30 key amino acid residues, of which Glu128, Arg34, Asp196, His368, Ser256, Glu25, Glu28, and Glu281 were the most common. In particular, Glu, Arg, and Asp appeared the most frequently. Amino acid residue Glu128 is the common binding site of umami peptides and receptors in all pigeon meat. This amino acid residue can form salt bridges, hydrogen bonds, electrostatic interactions, and hydrophobic interactions with 15 umami peptides. Asp196 also binds with most umami peptides through hydrogen bonds and other forces. This suggests that Glu128 and Asp196 play key roles in the interaction of the identified umami peptides with their receptors. Asp and Glu are usually involved in the binding of umami peptides to T1R1/T1R3, which is consistent with the results of present studies.

Dang et al [28] confirmed similar results, namely that amino acid residues, such as Glu, are the main binding sites for the recognition of umami peptides in T1R3 receptors. The bitter amino acid residue Arg34 was also the main binding site for most umami peptides, indicating that it may contribute to the activity of umami peptides. Wang et al [29] determined that bitter amino acid residues, such as Arg, Leu, and Phe, can bind to the amino acid residues of the T1R3 receptor through interactions. It has been suggested that the bitter amino acid residues of the umami peptide may also contribute to its activity of umami peptide, similar to the results of our study. Therefore, it is confirmed that Glu128, Arg34, and Asp196 are the major amino acid residues that bind to the receptors and contribute significantly to the umami activity of umami peptides.

## CONCLUSION

In our study, the taste activity of umami peptides was predicted and evaluated by the enzymolysis of polypeptides in pigeon meat, and umami peptides were identified by isolation and purification. The molecular docking of 15 identified umami peptides showed that umami peptides could bind to T1R1/T1R3, Glu128, and Asp196, which play a key role in the formation of umami.

Analysis of the binding site of the peptide and T1R1/T1R3 revealed that the binding site mainly involves four forces: salt bridges, hydrogen bonds, hydrophobic interactions, and electrostatic interactions. This study reveals the source and freshening mechanism of umami substances in pigeon meat and provides technical support for the discovery of new umami peptides from poultry resources.

## Figures and Tables

**Table 1:** Distribution of determination of molecular weight

	Molecular weight range (Da)	Content (%)	Number-average molecular weight (Mn)	Weight-average molecular weight (Mw)
Umami peptide base (GR)	>3,000	11.16	5,272	7,087
	1,000–3,000	23.88	1,522	1,667
	500–1,000	24.41	648	671
	189–500	27.93	258	293
	<189	12.62	-	-

The molecular weight of the enzymolysis polypeptide crude extract was mainly distributed in the range of less than 3,000 Da.

GR, crude extract of pigeon meat umami peptide without enzymatic hydrolysis; Mn, the average molecular weight is the average molecular weight calculated according to the number of molecules or the amount of substances; Mw, weight average molecular weight is the average molecular weight of the polymer according to the mass statistics.

**Table 2:** Prediction of taste activity in target peptides

Peptides	Frequency of active fragment occurrence							

	Astringent	Bitter taste	Bitter inhibition	Saline taste	Tart flavour	Sweet taste	Sweet inhibition	Umami flavour
TKQEYDEAGPS	0.0909	0.4545	0.0909	0.1818	0.4545	0.3636	-	0.5455
ITKQEYDEAGPS	0.0833	0.4167	0.0833	0.1667	0.4167	0.3333	-	0.5000
IHAEAPEFVEMS	-	0.5833	-	-	0.4167	0.3333	-	0.6667
TDADAMKYT	0.1111	0.4444	0.1111	0.2222	0.3333	0.3333	-	0.6667
LTDADAMKYT	0.1	0.5000	0.1000	0.2000	0.3000	0.3000	-	0.6000
LNAEGSVEEVFQQ	-	0.6923	0.1538	0.0769	0.3846	0.3846	0.0769	0.7692
KAFEEAEK	0.2500	0.7500	0.3750	0.1250	0.6250	0.5000	0.1250	0.8750
IDDLEDELYAQK	0.0833	0.7500	0.1667	0.5833	0.8333	0.1667	-	0.9167
ISEELDHALNDMT	-	0.3846	0.0769	0.2308	0.3846	0.0769	0.0769	0.6154
YQSEEDRKNIL	0.0909	0.4545	0.3636	0.3636	0.4545	0.0909	0.0909	0.6364
IYGAEARKESRG	0.0833	0.5000	0.3333	-	0.2500	0.4167	-	0.5833
IKWADAGAEYVVE	0.0769	0.6923	0.0769	0.0769	0.3846	0.6154	-	0.8462
TDKLKEAETRAE	0.1667	0.4167	0.25	0.0833	0.5	0.3333	-	0.6667
LTYQSEEDRKNI	0.0833	0.3333	0.3333	0.3333	0.4167	0.0833	0.0833	0.5833
LGDEETVRKAMEA	0.0769	0.5385	0.2308	0.3077	0.5385	0.3846	0.0769	0.6923

The values in the table refer to the frequency of occurrence of umami peptides, which is calculated by the formula A = a/N. a represents the number of fragments with specific taste activity in the target peptide sequence, and N represents the amino acid residue in the target peptide.

-, indicates the non-taste active fragment of the target peptide sequence; A, alanine; C, cysteine; D, aspartic acid; E, glutamic acid; F, phenylalanine; G, glycine; H, histidine; I, isoleucine; K, lysine; L, leucine; M, methionine; N, asparagine; P, proline; Q, glutamine; R, arginine; S, serine; T, threonine; W, tryptophan; V, valine; Y, tyrosine.

**Table 3:** The docking energy of umami peptide and umami receptor T1R1/T1R3

Peptides	Length	Total docking energy (kcal/mol)	Docking interaction energy (kcal/mol)
TKQEYDEAGPS (R-1)	11	−92.6761	−68.0350
ITKQEYDEAGPS (R-2)	12	−133.901	−85.0137
IHAEAPEFVEMS (R-3)	12	−139.338	−86.8868
TDADAMKYT (R-4)	9	−148.686	−66.9112
LTDADAMKYT (R-5)	10	−156.663	−94.9048
LNAEGSVEEVFQQ (R-6)	13	−160.730	−82.6129
KAFEEAEK (R-7)	8	−162.276	−95.2294
IDDLEDELYAQK (R-8)	12	−162.702	−73.5547
ISEELDHALNDMT (R-9)	13	−166.495	−83.3791
YQSEEDRKNIL (R-10)	11	−170.372	−93.6357
IYGAEARKESRG (R-11)	12	−179.531	−109.370
IKWADAGAEYVVE (R-12)	13	−180.501	−129.470
TDKLKEAETRAE (R-13)	12	−187.449	−112.367
LTYQSEEDRKNI (R-14)	12	−188.445	−120.456
LGDEETVRKAMEA (R-15)	13	−241.395	−159.388

The lower the binding energy, the more stable the acceptor-ligand complex.

**Table 4:** Key binding sites of umami peptides to umami receptors T1R1/T1R3

Binding site	R1	R2	R3	R4	R5	R6	R7	R8	R9	R10	R11	R12	R13	R14	R15
Glu128	*	3*	5*	*	^**^	5*	3*	*	^**^	3*	3*	4*	^**^	5*	2*
Arg34	*	3*	^**^	*	4*	*	^**^		3*	^**^	*	^**^	*	*	4*
Asp196	3*	*	*	*		^**^	*			^**^	*	5*		^**^	3*
His368	*		4*		4*			*	^**^	*	*	^**^		3*	
Ser256				*		^**^	*	*	*	*		^**^			3*
Glu25			*		3*			*		3*	3*		^**^	^**^	
Glu28				*				*		^**^	*	*		*	
Glu281				*			4*			^**^	*	*	*		
His125					*			*	*				*	^**^	*
Asn48		*	*					*			^**^				*
Asp170	*	*					^**^		*		*				
Glu197	^**^										*	^**^		^**^	*
Thr285		*							*		^**^	*			3*
Pro86			*		^**^							^**^		*	
Ser84	*		*		*								^**^		
Ser126			*		^**^				*					*	
Ser127		*			*		*							*	
Arg32		^**^						*				^**^			
Arg227		*						^**^		*					*
Glu85					*								^**^	*	
Leu284			*			*				*					
Met131			*		*							*			
Ser150		*					*				*				
Tyr198	*			*			*								
Val132			*		*							^**^			
Val257		*				^**^		*							
Leu365												*		*	
Lys135													*	*	
Lys437												^**^			*
Ser47			^**^					*							

During the interaction between umami peptides and receptors, there were 30 key amino acid residues, mainly including Glu128, Arg34, Asp196, His368, Ser256, Glu25, Glu28 and Glu281, among which Glu, Arg and Asp appeared most frequently.

Glu, glutamic acid; Arg, arginine; Asp, aspartic acid; His, histidine; Ser, serine; Asn, asparagine;Thr, threonine; Pro, proline; Leu, leucine; Met, methionine; Tyr, tyrosine; Val, valine; Lys, lysine.

* Indicates the number of ligand-receptor interactions.
